# Invasive Renal Parenchymal Urothelial Carcinoma: A Case Report and Literature Review of Imaging Features and Spectrum of Infiltrative Renal Masses

**DOI:** 10.7759/cureus.108880

**Published:** 2026-05-15

**Authors:** Sonia Loughraieb, Rania Guendouzi, Fathi Terki, Khadidja Mokeddem, Chaffa Aimeur

**Affiliations:** 1 Radiology Department, Mustapha University Hospital Center, Algiers, DZA; 2 Urology Department, Mustapha University Hospital Center, Algiers, DZA; 3 Pathology Department, Djilali Bounaama Douera University Hospital, Algiers, DZA

**Keywords:** diagnostic imaging, infiltrative renal masses, multiphasic contrast-enhanced ct, renal cell carcinoma, upper tract urothelial carcinoma

## Abstract

Upper tract urothelial carcinoma (UTUC) is a rare and aggressive malignancy. When UTUC invades the renal parenchyma, its imaging appearance may overlap with other infiltrative renal masses (IRMs), including renal cell carcinoma (RCC), renal lymphoma, metastases, and inflammatory or infectious lesions.

A 58-year-old female patient presented with painless gross hematuria. Her medical history included a right renal cyst that was previously aspirated, with no further details available. An ultrasound was performed, but it was inconclusive. A contrast-enhanced CT demonstrated a large, diffusely hypoenhancing infiltrative tumor in the mid and upper poles of the right kidney, with largely preserved renal contour. The mass extended into the renal pelvis. The imaging findings were suggestive of an invasive renal parenchymal urothelial carcinoma (UC). A decision was made not to perform a biopsy, and the patient subsequently underwent a nephroureterectomy with extended lymph node dissection. Histopathological examination confirmed the diagnosis of UC.

This case highlights the importance of recognizing invasive renal parenchymal UC as part of the spectrum of IRMs, which may represent an initial diagnostic clue. It also emphasizes the need to accurately distinguish UC from other IRMs, given the significant differences in management.

## Introduction

Upper tract urothelial carcinoma (UTUC) arises from the renal pelvis or ureter and typically affects elderly patients. It accounts for approximately 5%-10% of all renal tumors. Clinically, hematuria is the most common presenting symptom, which may be macroscopic or microscopic. Most renal masses are solitary, well-circumscribed, and encapsulated [1]. In contrast, invasive renal parenchymal UC is classified within the infiltrative renal masses (IRMs), which are enhancing renal lesions with an irregular shape and a poorly defined interface with the surrounding normal renal parenchyma. Renal cell carcinoma (RCC) is the most frequent IRM, accounting for about two-thirds of cases, followed by UC and lymphoma. Together, these three entities represent the vast majority of IRMs [2].

Contrast-enhanced CT is the primary imaging modality for evaluating the urinary tract. The most useful initial CT feature for identifying invasive renal parenchymal UC is, as a first step, classifying the lesion within the spectrum of IRMs based on its infiltrative growth pattern. Additional important findings include a filling defect in the renal pelvis, a tumor centered within the collecting system, absence of necrotic change, and mild post-contrast enhancement. Differentiation of invasive renal parenchymal UC from other IRMs is often challenging, but it is essential because management strategies differ significantly [3,4].

In this case report, we highlight the importance of recognizing the imaging features of invasive renal parenchymal UC, in which its recognition as part of the spectrum of IRMs may represent the first diagnostic clue. It also emphasizes the need to identify UC among other IRMs, as management differs significantly, ranging from systemic chemotherapy in lymphoma, to partial or radical nephrectomy in RCC, and up to radical nephroureterectomy (RNU) with bladder cuff excision in UC.

## Case presentation

A 58-year-old female presented with a three-day history of macroscopic hematuria associated with right lower back pain. Blood count was normal, with no evidence of anemia. The patient reported a known right kidney cyst, an incidental finding on a prior image examination, that was previously aspirated, but no additional details were available. She had no history of smoking or alcohol intake. Her medical and surgical history was insignificant, and she had no family history of cancer. On examination, the patient was conscious, and her vitals were stable. Systemic examination was essentially normal. Laboratory investigations revealed normal creatinine and urea levels and the presence of blood and trace leukocytes on urinalysis. Urine cytology was negative for high-grade UC.

A renal ultrasound was performed but was inconclusive. It demonstrated poor corticomedullary differentiation in the upper half of the right kidney. A mid-polar exophytic cortico-medullary cyst measuring 30 × 22 mm was also identified, with peripheral renal calcifications noted adjacent to the cyst. No hydronephrosis or vesical blood retention was observed.

**Figure 1:**
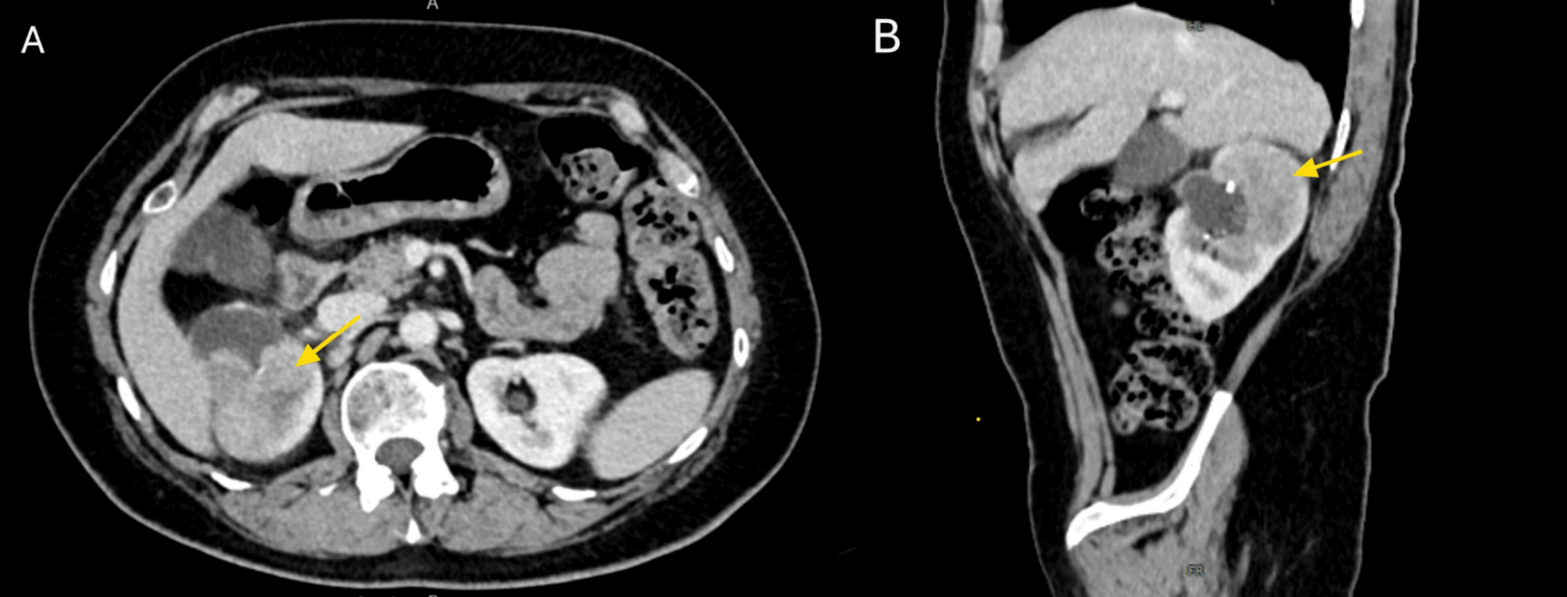
Contrast-enhanced nephrographic-phase axial (A) and sagittal (B) images An infiltrative, homogeneously enhancing mass (arrows) in the right kidney, affecting mid and upper poles, with preservation of the renal contour.

Subsequently, a multiphasic contrast-enhanced CT scan of the abdomen and pelvis was performed, given that CT is the primary imaging modality for a complete evaluation of the urinary tract. The right kidney was normal in size and globular, measuring 100 × 60 mm. It contained a hypovascular tumor mass involving the mid and upper renal parenchyma, sparing only the inferior pole. The lesion was spontaneously isodense (38 HU) and demonstrated mild, homogeneous enhancement on the nephrographic phase (76 HU), measuring approximately 60 × 50 × 40 mm (Figure 1).

**Figure 2:**
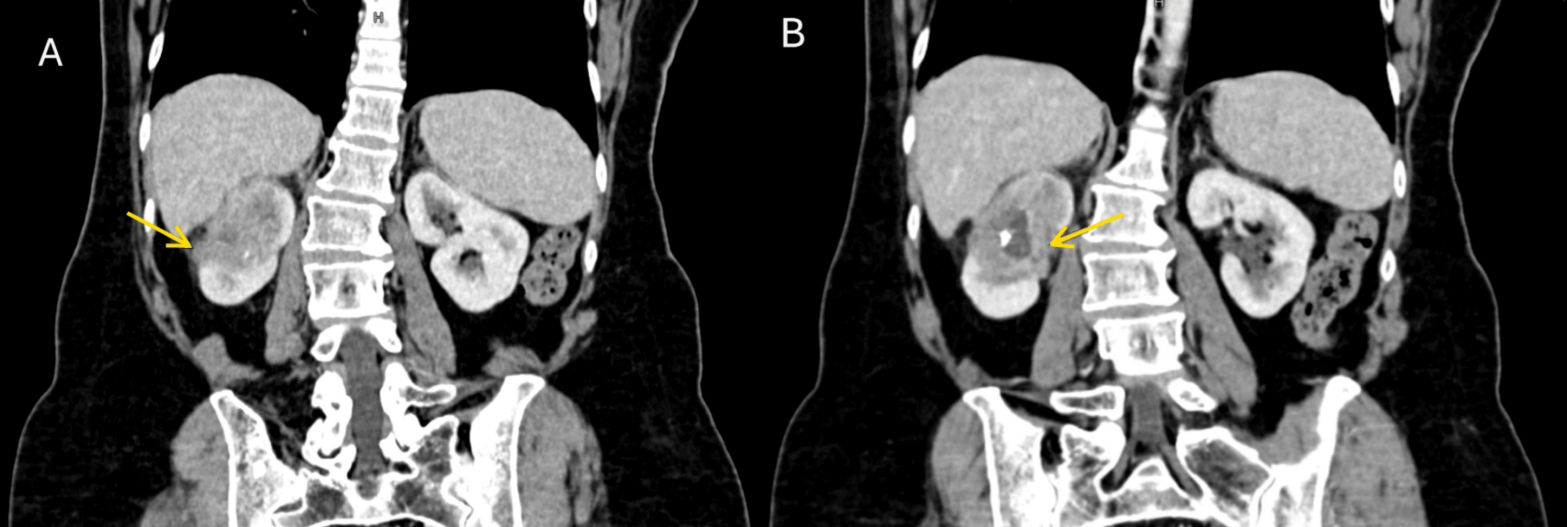
Contrast-enhanced nephrographic phase coronal images A: Mild focal contour change (arrow). B: Tumor mass extends into the renal pelvis (arrow).

The interface between the mass and the adjacent normal parenchyma was poorly defined. The renal contour remained preserved, except for small focal bulging along the mid-lateral aspect (Figure 2).

**Figure 3:**
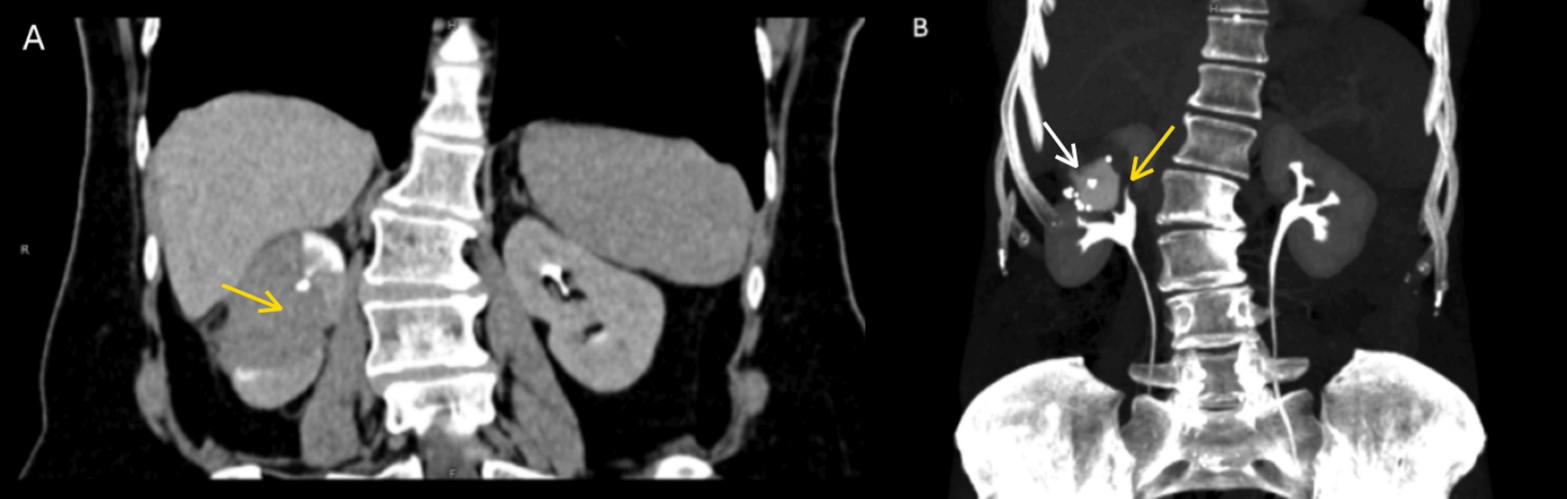
Axial CT urography A: The lesion extends into the renal sinus fat (arrow). B: Filling defect involving the middle and upper calyceal groups (yellow arrow), along with a known contrast-filled cyst fistulized to the excretory system (white arrow).

The mass involved the middle and upper calyceal groups, appearing as a filling defect on the excretory phase. It extended into the renal sinus fat and was associated with smooth thickening of the renal pelvis and proximal ureter (Figure 3). 

The mass abutted a previously aspirated mid-polar cyst, which appeared fistulized to the excretory system, seen as a contrast-filled outpouching measuring 25 × 30 mm. Hyperdense calcifications were noted at the periphery of the cyst.

The renal pedicle was single and patent, consisting of one renal vein and one renal artery, the latter showing early division approximately 15 mm from its origin. There was no evidence of renal pedicle or inferior vena cava involvement. Two right hilar lymph nodes were noted, measuring up to 5 mm in short axis.

No synchronous ureteral or bladder lesions were detected. There was no evidence of metastatic disease to the adrenal glands, contralateral kidney, liver, or bones.

**Figure 4:**
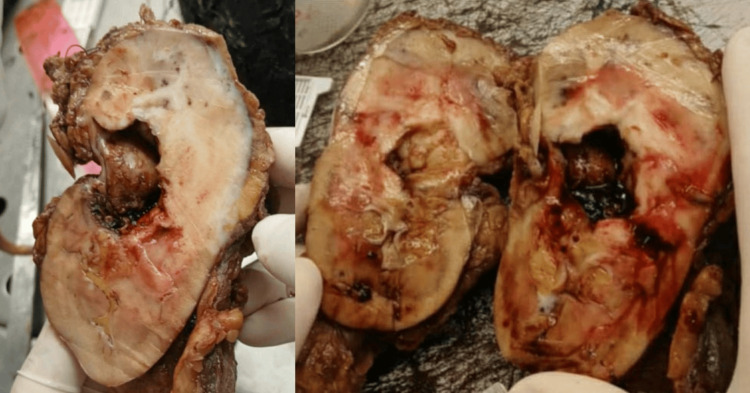
Gross specimen of the resected right renal mass following radical nephroureterectomy Demonstrating an infiltrative, whitish, friable, ill-defined mass, consistent with the preoperative imaging findings.

Based on the imaging findings, the diagnosis was suggestive of invasive renal parenchymal UC, and the patient underwent RNU with bladder cuff excision and lymphadenectomy (Figure 4). No preoperative biopsy was performed.

**Figure 5:**
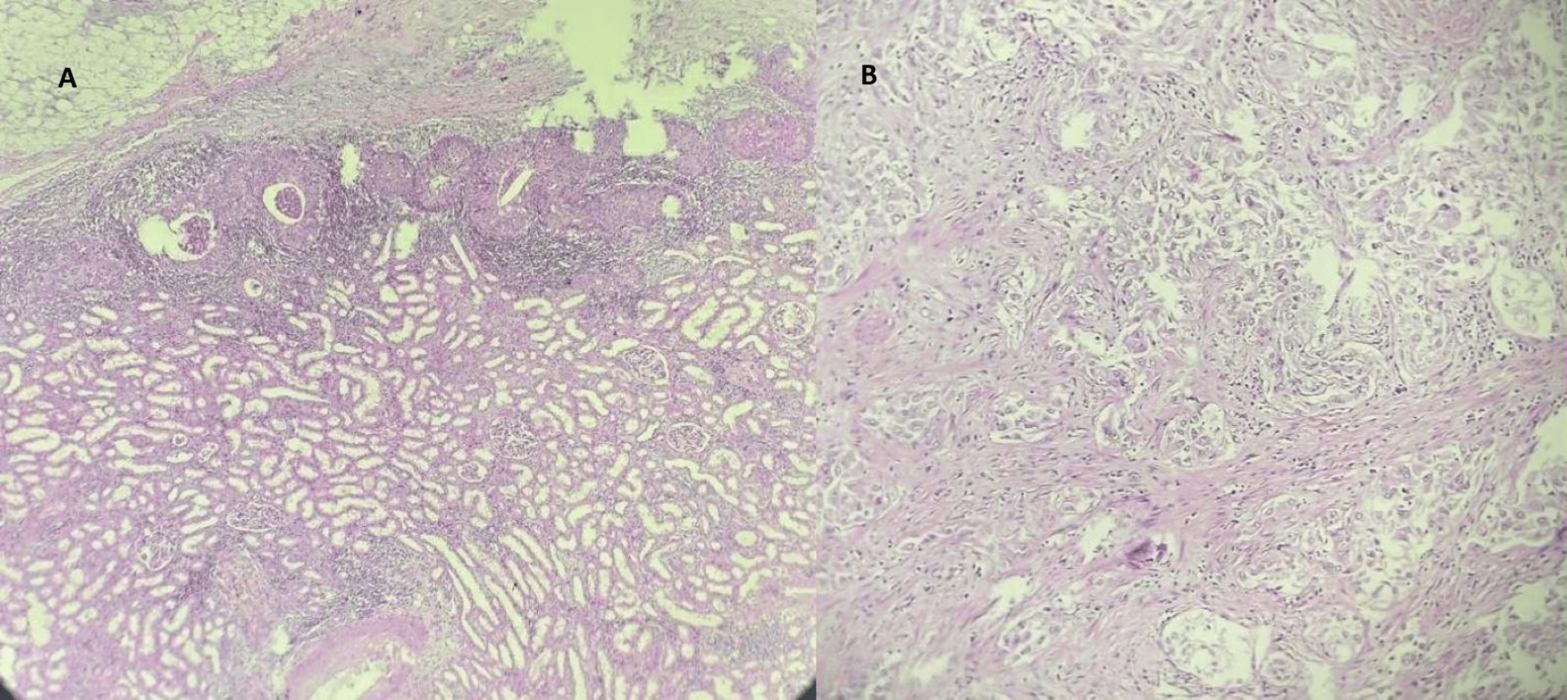
Histopathological examination A: Renal parenchyma extensively infiltrated by a malignant tumor proliferation, composed of urothelial nests and cords (H&E, ×200). B: The tumor nests are composed of urothelial cells showing moderate to marked cytonuclear atypia, with focal areas of necrosis (H&E, ×400).

Histopathological examination showed papillary structures of neoplastic urothelium arising from the pyelocaliceal system, with high-grade features invading the renal parenchyma, which was extensively infiltrated by tumor nests and tubules, demonstrating a poorly defined interface with the renal parenchyma and entrapment of normal structures. These findings were consistent with a high-grade invasive renal UC with squamous differentiation of the upper urinary tract, staged pT4N0Mx (Figure 5). The patient remains disease-free on follow-up and is under close surveillance due to the high risk of recurrence.

## Discussion

UC or transitional cell carcinoma is the fourth most common type of cancer. About 90%-95% of cases originate in the lower urinary tract, mainly affecting the bladder. The remaining 5%-10% arise in the upper urinary tract, most frequently in the pelvicalyceal system, followed by the ureter. UTUC represents approximately 5-10% of all renal tumors [1,5].

Clinically, these tumors occur most frequently in patients aged 70-90 years and with a male-to-female ratio of approximately 2:1 [1]. The most common clinical manifestation is hematuria, either macroscopic or microscopic, occurring in 70-80% of patients. In accordance with the literature, our patient presented for medical consultation due to gross hematuria. Flank pain is observed in 20-40% of cases, while a lumbar mass is found in 10-20% of patients [5].

Invasive renal parenchymal UC is considered part of the spectrum of IRMs. By definition, IRMs are enhancing renal lesions with an irregular shape and a poorly defined interface with the normal parenchyma in at least one unequivocal area. These tumors have been considered relatively uncommon, estimated to represent about 1% of all renal tumors. However, recent data suggest that IRMs are likely underrecognized rather than truly rare, as the infiltrative pattern is often not reported in preoperative imaging [2]. During imaging evaluation, we identified the right renal mass as an IRM, which constituted the first step toward suggesting UC. Histologically, these tumors are characterized by the interstitial proliferation of malignant cells around nephrons, collecting ducts, and vascular structures, allowing them to infiltrate the renal parenchyma without forming a distinct, well-defined mass [6]. 

The identification and characterization of IRMs have become a critical focus in urological radiology following the 2018 Society of Abdominal Radiology guidelines, which recommend routine documentation of whether a mass is circumscribed or infiltrative, although this decision was not unanimous, and many radiologists and urologists did not believe that this information is essential [7,8]. On the other hand, some reports have supported the importance of accurately identifying the infiltrative growth pattern, which is considered an independent negative prognostic factor regardless of histologic subtype [2,3]. 

Invasive renal parenchymal UC accounts for approximately 18% of IRMs. RCC is the most common at about 68%, including a predominance of grade 4 and poorly differentiated histologies (e.g., collecting duct, renal medullary, and unclassified RCC). Lymphoma represents 7.4% of cases. Together, these three entities constitute nearly 93% of all infiltrative renal lesions [2,6].

Contrast-enhanced CT is increasingly used for a complete evaluation of the urinary tract, particularly in patients presenting with hematuria. For renal lesion characterization, a dedicated imaging protocol is used, including four phases: an unenhanced phase, a corticomedullary phase at 35 seconds, a nephrographic phase at 90 seconds, and an excretory phase beginning 180 seconds after contrast administration. However, the nephrographic phase remains the most commonly used sequence for assessing detected renal masses [3,6]. Optimal opacification and distention of the urinary tract are achieved with oral hydration and the administration of furosemide prior to the examination [1]. 

The main diagnostic challenge is differentiating invasive renal parenchymal UC from other types of IRMs. Unlike RCC, which typically distorts the kidney's contour through expansile growth, roughly 90% of infiltrative UC cases maintain a normal renal outline [4]. However, in our case, there was a small focal bulge along the mid-lateral aspect of the kidney, with the rest of the renal contour being preserved. The other most useful diagnostic signs for identifying invasive renal parenchymal UC include a filling defect in the collecting system and a tumor center perceived to be within the collecting system. Furthermore, it typically appears as an endophytic and solid mass that is free from necrotic changes, which are hallmark features of aggressive RCC subtypes [3,6]. On contrast-enhanced CT, UC typically shows poor, homogeneous enhancement compared to the pronounced enhancement of RCC [9]. In our case, all of these characteristic imaging features of UC were present.

Within the differential diagnosis of invasive renal parenchymal UC, certain rare subtypes of RCC should be considered separately, particularly collecting duct carcinoma and renal medullary carcinoma. Collecting duct carcinoma arises from the renal medulla and typically presents as a centrally located, infiltrative mass with ill-defined margins that may extend into the renal sinus or pelvis, closely mimicking UC on imaging due to its hypovascular and non-expansile growth pattern. In older pathology literature, it has sometimes been called Bellini duct carcinoma, reflecting a presumed origin from the distal collecting ducts of Bellini; however, the exact cell of origin remains debated [10,11]. Renal medullary carcinoma, while sharing a similar medullary origin and aggressive infiltrative appearance, is distinguished by its strong association with sickle cell trait and its predilection for younger patients and often involves the right kidney [12].

Beyond RCCs, renal lymphoma represents a significant type of IRMs. It commonly appears as a large, uniform mass with little necrosis and may affect one or both kidneys. The disease can result from hematogenous dissemination or direct spread from retroperitoneal lymph nodes. Primary isolated renal lymphoma is very uncommon, accounting for under 1% of all extranodal lymphomas [6,13]. In patients with a known history of malignancy, metastases are the most common cause. Medial extension of a lesion into the hilar region, or a primary origin in this area, is relatively uncommon in cases of renal metastatic disease. Infiltrative, inflammatory, or infectious conditions may further mimic malignant IRMs on imaging [2,8].

Therapy planning for IRMs depends on an accurate diagnosis. UTUC is managed with RNU and bladder cuff excision due to a high risk of recurrence and synchronous UCs in the urinary tract, while centrally located RCC is treated with nephrectomy, often using minimally invasive techniques [3]. Renal lymphoma, in contrast, is treated primarily with systemic chemotherapy, while radiotherapy is reserved for selected cases [13]. When imaging is inconclusive, percutaneous or endoscopic biopsy is recommended to confirm the diagnosis and guide appropriate therapy [6]. In the present case, a multidisciplinary team decided not to perform a biopsy and proceeded with RNU, as the clinical and imaging features were highly suggestive of UC.

## Conclusions

Invasive renal parenchymal UC is a rare but important entity within the spectrum of IRMs. Accurate recognition of its key imaging features is essential for appropriate diagnosis and management. This case highlights the importance of identifying IRMs as an initial diagnostic step in suggesting UC and emphasizes the role of multiphasic CT in differentiating UC from other IRMs, thereby guiding optimal therapeutic management.
